# Prognostic Value of Drinking Status and Aldehyde Dehydrogenase 2 Polymorphism in Patients With Head and Neck Squamous Cell Carcinoma

**DOI:** 10.2188/jea.JE20140240

**Published:** 2016-06-05

**Authors:** Daisuke Kawakita, Isao Oze, Satoyo Hosono, Hidemi Ito, Miki Watanabe, Yasushi Yatabe, Yasuhisa Hasegawa, Shingo Murakami, Hideo Tanaka, Keitaro Matsuo

**Affiliations:** 1 Division of Epidemiology and Prevention, Aichi Cancer Center Research Institute, Nagoya, Japan; 1 愛知県がんセンター研究所 疫学・予防部; 2 Department of Otorhinolaryngology, Head and Neck Surgery, Nagoya City University Graduate School of Medical Sciences, Nagoya, Japan; 2 名古屋市立大学大学院 耳鼻咽喉・頭頸部外科; 3 Department of Pathology and Molecular Diagnosis, Aichi Cancer Center Hospital, Nagoya, Japan; 3 愛知県がんセンター中央病院 遺伝子病理診断部; 4 Department of Head and Neck Surgery, Aichi Cancer Center Hospital, Nagoya, Japan; 4 愛知県がんセンター中央病院 頭頸部外科; 5 Department of Epidemiology, Nagoya University Graduate School of Medicine, Nagoya, Japan; 5 名古屋大学大学院医学研究科 疫学; 6 Division of Molecular Medicine, Aichi Cancer Center Research Institute, Nagoya, Japan; 6 愛知県がんセンター研究所 遺伝子医療研究部

**Keywords:** alcohol drinking, ALDH2, head and neck cancer, squamous cell carcinoma, survival, アルコール飲酒, ALDH2, 頭頸部癌, 扁平上皮癌, 予後

## Abstract

**Background:**

The association between alcohol drinking, aldehyde dehydrogenase 2 (*ALDH2*) polymorphism, and survival in patients with head and neck squamous cell carcinoma (HNSCC) remains unclear.

**Methods:**

We performed a retrospective cohort study of 267 HNSCC patients at Aichi Cancer Center. Of these, 65 patients (24%) were non-drinkers, 104 (39%) were light drinkers (ethanol <46 g or <5 days/week), 46 (17%) were moderate drinkers (ethanol intake 46–68 g/day and ≥5 days/week), and 52 (20%) were heavy drinkers (ethanol intake ≥69 g and ≥5 days/week). The prognostic value of pre-treatment drinking status and *ALDH2* polymorphism was investigated using multivariate proportional hazard models.

**Results:**

Drinking status was associated with disease-free survival (DFS) in HNSCC patients, with marginal statistical significance (5-year DFS: 67.9% [95% confidence interval {CI}, 53.8–78.4%] for non-drinkers, 57.6% [95% CI, 47.4–66.6%] for light drinkers, 46.1% [95% CI, 30.8–60.1%] for moderate drinkers, and 43.5% [95% CI, 29.3–56.9%] for heavy drinkers; *P* = 0.088). However, this association lost significance when multivariate analyses were adjusted for established prognostic factors. *ALDH2* genotype was not significantly associated with DFS in HNSCC patients (5-year DFS: 85.7% [95% CI, 53.9–96.2%] for Lys/Lys, 56.2% [95% CI, 47.4–64.1%] for Glu/Lys, and 50.5% [95% CI, 40.3–59.7%] for Glu/Glu; *P* = 0.154). After stratification by *ALDH2* genotype, we observed a significant positive dose-response relationship between drinking status and DFS in HNSCC patients with *ALDH2* Glu/Glu (*P*_trend_ = 0.029).

**Conclusions:**

In this study, we identified a significant positive dose-response relationship between pre-treatment drinking status and DFS in HNSCC patients with *ALDH2* Glu/Glu. To confirm this association, further study is warranted.

## ＜要旨＞

**＜背景＞:**

飲酒習慣とALDH2遺伝子多型の頭頸部癌予後への影響は明らかでない。

**＜方法＞:**

愛知県がんセンター中央病院を受診した頭頸部扁平上皮癌患者267例を対象に、後ろ向きコホート研究を実施した。飲酒習慣に関しては、65人(24%)が非飲酒者、104人(39%)が軽度飲酒者 (エタノール46g未満/日、頻度5日/週未満)、46人(17%)が中等度飲酒者(エタノール46g以上69g未満、頻度5日/週以上)、52人(20%)が重度飲酒者(エタノール69g以上、頻度5日/週以上)であった。治療前飲酒習慣とALDH2遺伝子多型の予後への影響は、Cox比例ハザードモデルを用いて評価した。

**＜結果＞:**

飲酒習慣は頭頸部扁平上皮癌患者の無病生存へ影響を示した[5年無病生存率: 非飲酒者;67.9%(53.8-78.4)、軽度飲酒者;57.6%(47.4-66.6)、中等度飲酒者;46.1%(30.8-60.1)、重度飲酒者;43.5%(29.3-56.9)、P=0.088]。しかしながら、その関係性は他の予後因子を調整した多変量解析では一致しなかった。またALDH2遺伝子多型は無病生存と有意な関連を示さなかった[5年無病生存率: Lys/Lys;85.7%(53.9-96.2)、Glu/Lys;56.2%(47.4-64.1)、Glu/Glu;50.5%(40.3-59.7)、P= 0.154]。ALDH2遺伝子多型に基づいて層別化解析を行うと、ALDH2 Glu/Gluの患者群では飲酒習慣は無病生存に有意な正の量反応関係を示した (P_trend_= 0.029)。

**＜結論＞:**

今回我々はALDH2 Glu/Gluの頭頸部扁平上皮癌患者において、治療前飲酒習慣と無病生存の間に有意な正の量反応関係が存在することを示した。この関連を明らかにするためには、さらなる研究による検証が必要である。

## INTRODUCTION

Worldwide, almost 600 000 new cases of head and neck cancer are reported each year.^1^ Alcohol drinking is an established risk factor for head and neck squamous cell carcinoma (HNSCC).^2^^,^^3^ In general, ethanol is oxidized by alcohol dehydrogenase (ADH) enzymes to acetaldehyde, which is then further oxidized to acetate by aldehyde-dehydrogenase (ALDH) enzymes. This latter oxidation is largely dependent on the ALDH2 enzyme.^4^ The metabolism of ethanol leads to accumulation of acetaldehyde, which is toxic and established as a strong carcinogen, and differences in ethanol metabolism that result from polymorphisms in the genes that code for these enzyme affect cancer etiology among drinkers.^5^ The *ALDH2* Glu504Lys polymorphism (Single Nucleotide Polymorphism Database [dbSNP] ID, rs671), which has a catalytically inactive subunit, is known to interact with the association between alcohol drinking and HNSCC risk.^6^^–^^13^ Light drinkers with *ALDH2* Lys/Lys and Glu/Lys have 18 times and 5 times higher average peaks of acetaldehyde concentrations in blood, respectively, than moderate drinkers with *ALDH2* Glu/Glu.^14^ However, the prognostic value of alcohol drinking and *ALDH2* polymorphism on clinical outcome in patients with HNSCC remains unclear.^15^^–^^21^

Previously, we clarified the association between lifestyle factors and prognosis in HNSCC patients.^22^ Alcohol drinking is known to induce factors associated with cancer survival.^23^^–^^25^ This suggests that alcohol drinking might affect the survival of HNSCC patients and that *ALDH2* polymorphism might interact with this association.

Here, we conducted a retrospective cohort study to clarify the potential association of these factors among 267 patients treated for HNSCC at Aichi Cancer Center (ACC).

## METHODS

### Patients

We selected patients from the database of the Hospital-based Epidemiologic Research Program at Aichi Cancer Center (HERPACC), based at ACC in Nagoya, Japan. The HERPACC framework has been detailed elsewhere.^26^^,^^27^

Briefly, 23 408 HERPACC-enrolled, first-visit outpatients treated between January 2001 and November 2005 at ACC were asked to provide blood samples and information on lifestyle factors. Among those who participated, 22 727 patients (97.1%) completed a self-administered questionnaire including lifestyle factors, which was checked by a trained interviewer, and approximately 60% provided blood samples. The HERPACC study was approved by the Institutional Ethics Review Board of ACC, and all participants provided written informed consent.

HERPACC-enrolled patients diagnosed as having primary head and neck cancer who met the following criteria were included: (a) no prior history of cancer; (b) no malignant neoplasms of the salivary glands, nasopharynx, nasal, or paranasal sinuses (these cancers were excluded, as they have a distinct etiology); (c) histological diagnosis of squamous cell carcinoma; (d) Eastern Cooperative Oncology Group (ECOG) performance status (PS) of 0 to 2^28^; and (e) availability of information about drinking status and *ALDH2* genotype. Ultimately, 267 patients were eligible for this study.

### Treatment and follow-up

We considered surgery and chemoradiotherapy (CRT) or radiotherapy (RT) with or without induction chemotherapy (ICT) as treatment modalities for HNSCC. Attending surgeons determined treatment of each individual patient based on clinical disease stage, primary tumor site, and PS. Patients were followed up with a history and physical examination, complete blood cell count, and imaging examination every 3 to 6 months for 5 years after their treatment. We confirmed vital and disease status by checking medical records at the date of last follow-up visit. Vital status in patients lost to follow-up was confirmed by a census registration conducted annually.

### Genotyping of *ALDH2*

We extracted DNA from the buffy coat fraction with a DNA Blood mini kit (Qiagen, Tokyo, Japan) or BioRobot EZ1 and EZ1 DNA Blood 350 mL Kit (Qiagen). We genotyped *ALDH2* Glu504Lys polymorphism (rs671) by the TaqMan method (Applied Biosystems, Foster City, CA, USA). The quality control of genotyping was assessed statistically by using the Hardy-Weinberg test and by retyping of a random sampling of 5% of subjects.

### Assessment of drinking and smoking status

Details of the assessment of drinking and smoking status have been described elsewhere.^22^ Our questionnaire consisted of items related to smoking and drinking habits in the period preceding the development of the present symptoms or reason for visit to ACC.

Levels of alcohol consumption were divided into four groups: non-, light, moderate, and heavy drinkers. Moderate drinkers were defined as individuals who consumed alcoholic beverages in a daily amount of ≥46 g ethanol (equivalent to two Japanese drinks) but <69 g ethanol for ≥5 days per week. Heavy drinkers were defined as individuals who consumed alcohol beverages in a daily amount of ≥69 g ethanol for ≥5 days per week. Light drinkers were defined as individuals who consumed alcoholic beverages in a daily amount of <46 g ethanol for <5 days per week. The remaining patients were categorized as non- or light drinkers using data from the HERPACC study for head and neck cancers.^29^

Cumulative exposure to cigarette smoking was quantified as pack-years of smoking (PY), the product of the number of packs consumed per day and number of years of cigarette smoking. We divided patients into four groups based upon PY: non-, light (PY < 20), moderate (PY 20–39), and heavy smokers (PY ≥ 40).

### Statistical methods

The primary endpoint of this study was disease-free survival (DFS), which was defined as the number of days from the beginning of treatment to the date of relapse. The associations between drinking status (0: non, 1: light, 2: moderate, and 3: heavy), *ALDH2* genotypes (0: Glu/Lys, 1: Glu/Glu, and 2: Lys/Lys), and DFS were evaluated by the Kaplan-Meier product-limit method and uni- and multivariate Cox proportional hazards models. Confounders considered in the multivariate analyses were age (continuous), sex (male or female), ECOG PS (0–2), smoking status (non- vs light vs moderate vs heavy), Union for International Cancer Control (UICC) stage (1–4), and treatment method (surgery or CRT/RT).

In addition, the interaction between drinking status and *ALDH2* genotype was examined by adding an interaction term between the two items in multivariate models. Distribution of patient characteristics was assessed by the χ^2^ test or Fisher’s exact test, as appropriate. All statistical analyses were performed using the software STATA ver. 10 (Stata Corp, College Station, TX, USA). All tests were two-sided, and *P*-values of <0.05 were considered statistically significant.

## RESULTS

### Patient characteristics and survival

Table 1 summarizes subject characteristics in the study. Median age was 61 (range, 21–78) years, and median follow-up time was 5.0 years (range, 0.7 months–9.1 years). Drinking was more prevalent among males than females. Similarly, drinking was more prevalent among smokers, but less prevalent among oral cavity cancer patients than patients without oral cavity cancer. Five-year DFS among all patients was 55.5% (95% confidence interval [CI], 49.1–61.4%).

**Table 1.** Characteristics of head and neck squamous cell carcinoma patients according to drinking status and *ALDH2* genotype

Characteristics	Drinking status				*P*-value	*ALDH2* genotype (rs671)			*P*-value
	
	Non	Light	Moderate	Heavy		Glu/Glu	Glu/Lys	Lys/Lys	
	*n* = 65 (%)	*n* = 104 (%)	*n* = 46 (%)	*n* = 52 (%)		*n* = 111 (%)	*n* = 142 (%)	*n* = 14 (%)	
Median age (range)	61 (23–78)	60 (21–77)	61 (42–77)	61 (32–78)		61 (21–78)	61 (30–78)	56 (25–71)	
Sex									
Male	32 (49)	90 (87)	43 (93)	51 (98)	<0.001	82 (74)	122 (86)	12 (86)	0.048
Female	33 (51)	14 (13)	3 (7)	1 (2)		29 (26)	20 (14)	2 (14)	
ECOG PS									
0	29 (45)	55 (53)	26 (57)	27 (52)	0.491	59 (53)	71 (50)	7 (50)	0.922
1	33 (51)	43 (41)	19 (41)	25 (48)		48 (43)	65 (46)	7 (50)	
2	3 (4)	6 (6)	1 (2)	0 (0)		4 (4)	6 (4)	0 (0)	
UICC stage									
I	12 (19)	21 (20)	6 (13)	8 (15)	0.172	19 (17)	23 (16)	5 (36)	0.502
II	22 (33)	21 (20)	6 (13)	12 (23)		26 (23)	31 (22)	4 (29)	
III	12 (19)	18 (17)	10 (22)	6 (12)		21 (19)	23 (16)	2 (14)	
IV	19 (29)	44 (43)	24 (52)	26 (50)		45 (41)	65 (46)	3 (21)	
Primary tumor site									
Oral cavity	34 (52)	56 (54)	19 (41)	16 (31)	<0.001	61 (55)	58 (41)	6 (42)	0.003
Oropharynx	11 (17)	16 (15)	4 (9)	16 (31)		20 (18)	23 (16)	4 (29)	
Hypopharynx	3 (5)	15 (15)	11 (24)	15 (29)		8 (7)	36 (25)	0 (0)	
Larynx	17 (26)	17 (16)	12 (26)	5 (9)		22 (20)	25 (18)	4 (29)	
Treatment method									
Surgery	22 (34)	39 (37)	19 (41)	15 (29)	0.585	44 (40)	47 (33)	4 (29)	0.477
CRT or RT	43 (66)	65 (63)	27 (59)	37 (71)		67 (60)	95 (67)	10 (71)	
Smoking status									
Non	30 (46)	20 (19)	3 (6)	2 (4)	<0.001	28 (25)	22 (16)	5 (36)	0.217
Light (PY < 20)	7 (11)	21 (20)	9 (20)	4 (8)		19 (17)	20 (14)	2 (14)	
Moderate (20 ≤ PY < 40)	11 (17)	25 (24)	11 (24)	19 (36)		24 (22)	40 (28)	2 (14)	
Heavy (40 ≤ PY)	14 (21)	38 (37)	22 (48)	27 (52)		39 (35)	58 (41)	4 (29)	
Unknown	3 (5)	0 (0)	1 (2)	0 (0)		1 (1)	2 (1)	1 (7)	

ECOG, Eastern Cooperative Oncology Group; UICC, Union for International Cancer Control; PS, performance status; CRT, chemoradiotherapy; RT, radiotherapy; PY, pack-years of smoking; ALDH2; aldehyde dehydrogenase 2.

Drinking status was classified as follows: non, light (<46 g ethanol or <5 days/week), moderate (46–68 g ethanol for ≥5 days/week), and heavy (≥69 g ethanol for ≥5 days/week).

### Impact of drinking status and *ALDH2* genotype on DFS

**Figure 1.**
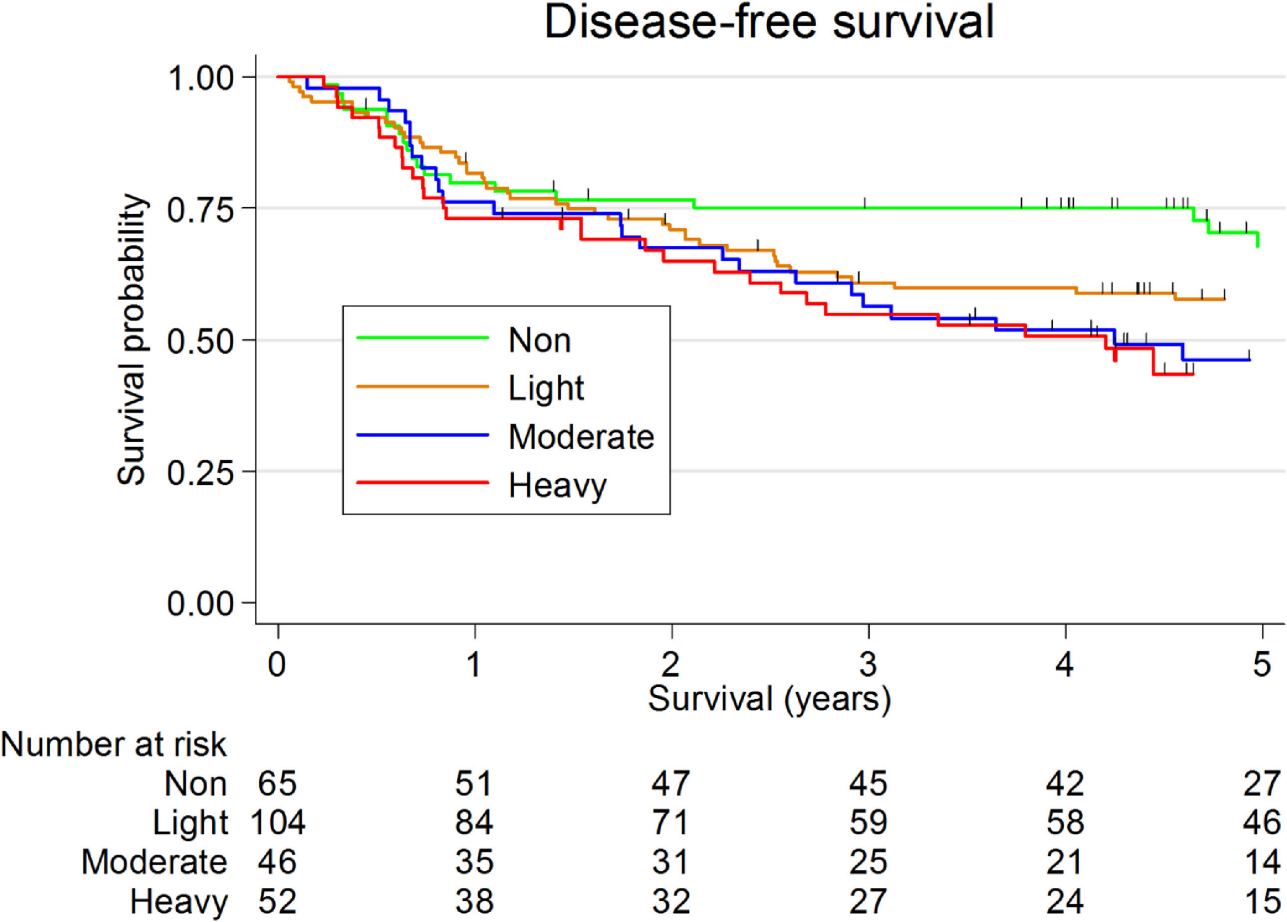
Kaplan Meier survival curves for drinking status in patients with head and neck squamous cell carcinoma. Five-year disease-free survival was 67.9% (95% confidence interval [CI], 53.8–78.4%) for non-drinkers, 57.6% (95% CI, 47.4–66.6%) for light drinkers, 46.1% (95% CI, 30.8–60.1%) for moderate drinkers, and 43.5% (95% CI, 29.3–56.9%) for heavy drinkers (logrank test, *P* = 0.088).

Figure 1 shows the Kaplan-Meier survival curves for drinking status. Drinking status was associated with DFS in HNSCC patients with marginal statistical significance (5-year DFS: 67.9% [95% CI, 53.8–78.4%] for non-drinkers, 57.6% [95% CI, 47.4–66.6%] for light drinkers, 46.1% [95% CI, 30.8–60.1%] for moderate drinkers, and 43.5% [95% CI, 29.3–56.9%] for heavy drinkers; *P* = 0.088).

**Figure 2.**
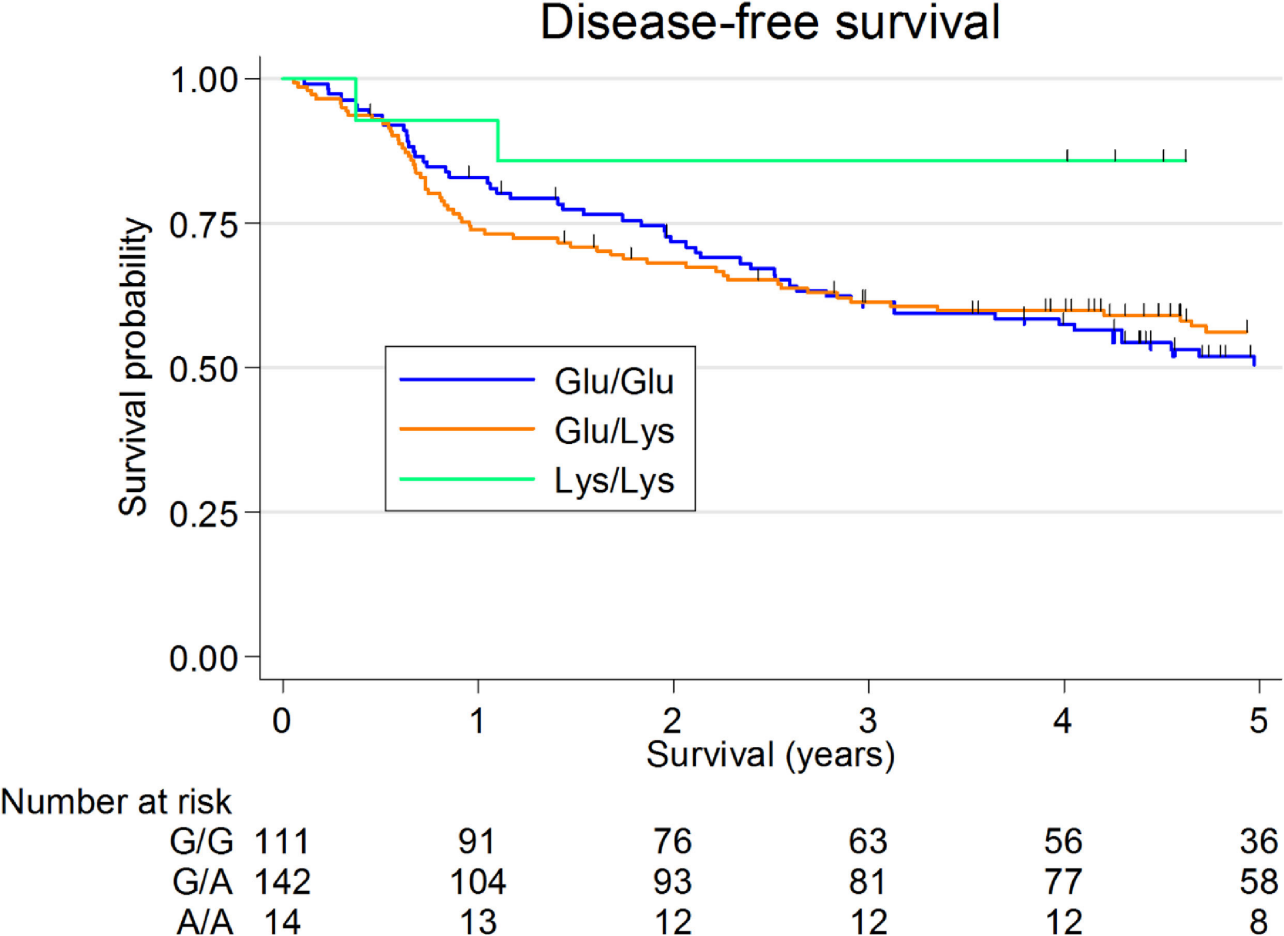
Kaplan Meier survival curves for aldehyde dehydrogenase 2 (*ALDH2*) genotype in patients with head and neck squamous cell carcinoma. Five-year disease-free survival was 85.7% (95% confidence interval [CI], 53.9–96.2%) for *ALDH2* Lys/Lys patients, 56.2% (95% CI, 47.4–64.1%) for *ALDH2* Glu/Lys patients, and 50.5% (95% CI, 40.3–59.7%) for *ALDH2* Glu/Glu patients (logrank test, *P* = 0.154).

Figure 2 shows the Kaplan-Meier survival curves for *ALDH2* genotype. *ALDH2* genotype was not significantly associated with DFS in HNSCC patients (5-year DFS: 85.7% [95% CI, 53.9–96.2%] for Lys/Lys, 56.2% [95% CI, 47.4–64.1%] for Glu/Lys, and 50.5% [95% CI, 40.3–59.7%] for Glu/Glu; *P* = 0.154).

Table 2 shows the results of uni- and multivariate analysis for DFS. In univariate analysis, drinking status showed a significant trend in decreasing DFS (*P*_trend_ = 0.013). However, neither drinking status nor *ALDH2* genotype showed a significant association with DFS in multivariate analysis.

**Table 2.** Impact of drinking status and *ALDH2* genotype on disease-free survival in patients with head and neck squamous cell carcinoma

Variable	*n*	*n* (relapse)	Univariate analysis			Multivariate analysis		
	
			HR	95% CI	*P*-value	HR	95% CI	*P*-value
Drinking status								
Non	65	16	1.00	reference	—	1.00	reference	—
Light	104	31	1.38	0.83–2.29	0.210	1.09	0.58–2.08	0.785
Moderate	46	20	1.80	1.02–3.18	0.042	1.32	0.64–2.71	0.453
Heavy	52	16	1.90	1.09–3.31	0.023	1.22	0.58–2.57	0.607
*P*_trend_					0.013			0.503

*ALDH2* genotype (rs671)								
Glu/Glu	111	39	1.00	reference	—	1.00	reference	—
Glu/Lys	142	41	0.98	0.68–1.40	0.914	0.76	0.51–1.13	0.178
Lys/Lys	14	3	0.34	0.11–1.08	0.067	0.51	0.14–1.84	0.303
*P*_trend_					0.212			0.123

ALDH2, aldehyde dehydrogenase 2; CI, confidence interval; HR, hazard ratio.

Drinking status was classified as follows: non, light (<46 g ethanol or <5 days/week), moderate (46–68 g ethanol for ≥5 days/week), and heavy (≥69 g ethanol for ≥5 days/week).

Adjusted for age, sex, performance status, clinical disease stage, primary tumor site, smoking, and definitive treatment.

### Interaction between drinking status and *ALDH2* genotype on DFS

**Figure 3.**
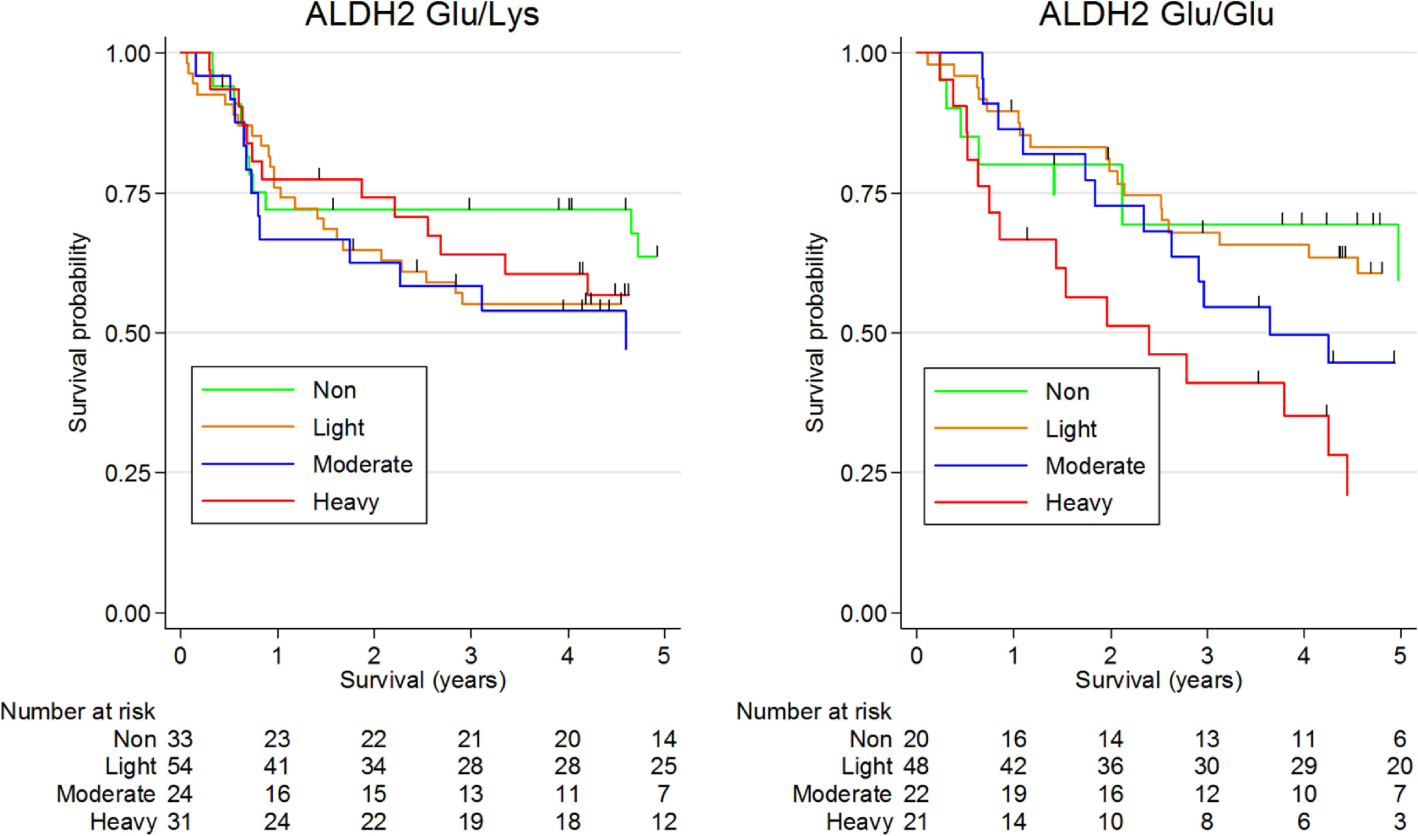
Kaplan Meier survival curves of disease-free survival for drinking status according to aldehyde dehydrogenase 2 (*ALDH2*) Glu/Glu genotype. Five-year disease-free survival among ALDH2 Glu/Lys patients was 63.6% (95% confidence interval [CI], 43.4–78.2%) for non-drinkers, 55.1% (95% CI, 40.8–67.3%) for light drinkers, 47.1% (95% CI, 25.2–66.3%) for moderate drinkers, and 56.8% (95% CI, 37.3–72.3%) for heavy drinkers (logrank test, *P* = 0.764). Respective rates among ALDH2 Glu/Glu patients were 59.4% (95% CI, 30.9–79.4%), 60.6% (95% CI, 44.8–73.2%), 44.6% (95% CI, 23.5–63.8%), and 21.1% (95% CI, 5.8–42.7%) (logrank test, *P* = 0.023).

Kaplan-Meier survival curves of DFS for drinking status according to *ALDH2* genotype are shown in Figure 3. Drinking status was significantly associated with DFS in *ALDH2* Glu/Glu patients (59.4% [95% CI, 30.9–79.4%] for non-drinkers, 60.6% [95% CI, 44.8–73.2%] for light drinkers, 44.6% [95% CI, 23.5–63.8%] for moderate drinkers, and 21.1% [95% CI, 5.8–42.7%] for heavy drinkers; *P* = 0.023).

In multivariate analysis, drinkers with *ALDH2* Glu/Glu showed a significant trend toward decreased DFS (*P*_trend_ = 0.029; Table 3). In contrast, among patients with *ALDH2* Glu/Lys, drinking status showed no significant association with DFS. In addition, we observed suggestive heterogeneity between drinking status and *ALDH2* genotype on DFS (*P* for heterogeneity = 0.100).

**Table 3.** Impact of drinking status according to *ALDH2* genotype on disease-free survival in patients with head and neck squamous cell carcinoma

*ALDH2* genotype (rs671)	Drinking status																					*P* for heterogeneity

	*n*	*n* (relapse)	Non			*n*	*n* (relapse)	Light			*N*	*N* (relapse)	Moderate			*N*	*N* (relapse)	Heavy			*P*_trend_	
			
			HR	95% CI	*P*-value			HR	95% CI	*P*-value			HR	95% CI	*P*-value			HR	95% CI	*P*-value		
Glu/Glu	20	5	1.13	0.42–3.01	0.814	48	13	1.05	0.48–2.31	0.895	22	11	1.77	0.73–4.29	0.204	21	10	2.34	0.93–5.86	0.070	0.029	0.138
Glu/Lys	33	9	1.00	reference	—	54	17	1.27	0.57–2.86	0.557	24	9	1.18	0.47–2.96	0.718	31	6	0.86	0.35–2.14	0.748	0.164	
Lys/Lys	12	2	0.49	0.10–2.27	0.359	2	1	2.00	0.22–18.04	0.535	0	0	—			0	0	—			—	

ALDH2, aldehyde dehydrogenase 2; HR, hazard ratio; CI, confidence interval.

Drinking status was classified as follows: non, light (<46 g ethanol or <5 days/week), moderate (46–68 g ethanol for ≥5 days/week), and heavy (≥69 g ethanol for ≥5 days/week).

Adjusted for age, sex, performance status, clinical disease stage, primary tumor site, smoking, and definitive treatment.

## DISCUSSION

In this study, we demonstrated that high pre-treatment alcohol consumption worsens DFS in HNSCC patients. This effect was evident only among the *ALDH2* Glu/Glu patients, suggesting that it might differ by *ALDH2* genotype. To our knowledge, this is the first report to evaluate the impact of alcohol drinking combined with *ALDH2* genotype on clinical outcome in HNSCC patients.

Although the mechanism behind this association between drinking status and survival of HNSCC patients remains unclear, several explanations appear plausible based on existing evidence. First, alcohol drinking may induce activation of NF-κB, a transcription factor that has been linked with the transformation of cells and survival of cancer stem cells.^30^ Additionally, NF-κB regulates the expression of genes associated with the apoptosis, proliferation, invasion, angiogenesis, and metastasis of cancer. Second, alcohol consumption may induce TP53 mutation, which is associated with HNSCC survival.^23^^,^^31^ Third, alcohol drinking may modify the DNA methylation profile in HNSCC cells. DNA methylation modifications may affect the prognosis of HNSCC.^32^ Fourth, alcohol drinking damages normal mucosa in the head and neck region. Long-term continuation of this mucosal damage, called “field cancerization”, may be associated with a predisposition to relapse or development of a second primary tumor (SPT).^25^ However, the incidence of SPT was not associated with drinking status in this study (data not shown).

In addition, our findings suggest that the association between alcohol drinking and prognosis of HNSCC may differ by *ALDH2* genotype. Although this mechanism is also unclear, we speculate that this association might be affected by alcohol dependence.^33^ Individuals who are heterozygous or homozygous for the Lys allele of *ALDH2* Glu504Lys polymorphism (rs671) have greatly reduced ability to metabolize acetaldehyde, which greatly decreases their risk for alcohol dependence.^34^ After definitive treatment, *ALDH2* Glu/Glu patients might maintain higher levels of alcohol consumption than *ALDH2* Glu/Lys patients. However, this point should be assessed in other studies evaluating post-treatment or under-treatment drinking behavior according to *ALDH2* genotype.

Our study has several methodological strengths. First, because clinicians involved in the care of study patients were not aware of the exposure status examined in this study, information bias was less likely to have been introduced. Second, because the analyses were adjusted for established prognostic factors, including clinical disease stage and PS, the observed associations were theoretically independent. However, the exclusion of residual confounding by unevaluated factors, such as human papilloma virus (HPV) infection, cannot be completely ruled out.

Several methodological limitations also warrant mention. First, our information on drinking habits reflected pre-treatment drinking status only, and we were therefore unable to evaluate the impact of changes in drinking behavior during the study. Second, the prognostic value of drinking-related comorbidities, including coronary artery disease, cerebral infarction, peripheral vascular disease, and chronic liver disease, might bias this study. If death induced from these comorbidities were not uncommon in the heavy drinkers, our study would have overestimated the prognostic value of heavy drinking in HNSCC patients. Actually, while the ratio of patients who died from HNSCC alone was 58.8%, information on the cause of death was unavailable for 29.5%. Third, although we tried to minimize the effect of bias by considering potential confounders in multivariate analysis, the impact of residual confounding, including that due to HPV infection, cannot be fully excluded. Finally, the moderate sample size may have limited the ability of the study to detect differences between the groups.

We concluded that high pre-treatment alcohol drinking worsened DFS in patients with HNSCC. This effect might be clearer among patients with *ALDH2* Glu/Glu. Our results suggest a possible gene-environmental interaction in the clinical outcome of HNSCC patients. Replication in a larger study is warranted.

## ONLINE ONLY MATERIAL

Abstract in Japanese.
