# Supplementary material for: Prognostic Value of Drinking Status and Aldehyde Dehydrogenase 2 Polymorphism in Patients With Head and Neck Squamous Cell Carcinoma
Source: J Epidemiol. 2016 Jun 5;26(6):292–9. doi: 10.2188/jea.JE20140240 (PMC4884897; doi:10.2188/jea.JE20140240)
Supplement: Abstract in Japanese. [file je-26-292-s001.pdf]

飲酒習慣と ALDH2 遺伝子多型の頭頸部がん予後への影響

川北 大介[1,2]、尾瀬 功[1]、細野 覚代[1]、伊藤 秀美[1]、渡邉 美貴[1]、谷田部 恭[3]
長谷川 泰久[4]、村上 信五[2]、田中 英夫[1,5]、松尾 恵太郎[5,6]
[1] 愛知県がんセンター研究所　疫学・予防部
[2] 名古屋市立大学大学院　耳鼻咽喉・頭頸部外科
[3] 愛知県がんセンター中央病院　遺伝子病理診断部
[4] 愛知県がんセンター中央病院　頭頸部外科
[5] 名古屋大学大学院医学研究科　疫学
[6] 愛知県がんセンター研究所　遺伝子医療研究部

＜要旨＞
＜背景＞
飲酒習慣と ALDH2 遺伝子多型の頭頸部癌予後への影響は明らかでない。
＜方法＞
愛知県がんセンター中央病院を受診した頭頸部扁平上皮癌患者 267 例を対象に、後ろ向きコホート研究を実施した。飲酒習慣に関しては、65 人(24%)が非飲酒者、104 人(39%)が軽度飲酒者 (エタノール 46g 未満/日、頻度 5 日/週未満)、46 人(17%)が中等度飲酒者(エタノール 46g 以上 69g 未満、頻度 5 日/週以上)、52 人(20%)が重度飲酒者(エタノール 69g 以上、頻度 5 日/週以上)であった。治療前飲酒習慣と ALDH2 遺伝子多型の予後への影響は、Cox 比例ハザードモデルを用いて評価した。
＜結果＞
飲酒習慣は頭頸部扁平上皮癌患者の無病生存へ影響を示した[5 年無病生存率: 非飲酒者;67.9%(53.8-78.4)、軽度飲酒者;57.6%(47.4-66.6)、中等度飲酒者;46.1%(30.8-60.1)、重度飲酒者;43.5%(29.3-56.9)、P=0.088]。しかしながら、その関係性は他の予後因子を調整した多変量解析では一致しなかった。また ALDH2 遺伝子多型は無病生存と有意な関連を示さなかった[5 年無病生存率： Lys/Lys;85.7%(53.9-96.2)、Glu/Lys;56.2%(47.4-64.1)、Glu/Glu;50.5%(40.3-59.7)、P= 0.154]。ALDH2 遺伝子多型に基づいて層別化解析を行うと、ALDH2 Glu/Glu の患者群では飲酒習慣は無病生存に有意な正の量反応関係を示した (P_{trend}= 0.029)。
＜結論＞
今回我々は ALDH2 Glu/Glu の頭頸部扁平上皮癌患者において、治療前飲酒習慣と無病生存の間に有意な正の量反応関係が存在することを示した。この関連を明らかにするためには、さらなる研究による検証が必要である。

キーワード:　アルコール飲酒、ALDH2、頭頸部癌、扁平上皮癌、予後
